# Supplementary material for: Are carbon emissions trading and green financial instruments synergistic? -Comprehensive quantitative research based on content analysis
Source: PLoS One. 2024 Mar 7;19(3):e0298601. doi: 10.1371/journal.pone.0298601 (PMC10919601; doi:10.1371/journal.pone.0298601)
Supplement: S1 Data — (ZIP) [file pone.0298601.s001.zip › Data/地级市Z_F工作报告.zip!/║╙▒▒/│╨╡┬╩╨/└┤╫╘╣1⁄2╓┌║┼í╛┬φ┐╦╩2╛▌═°í┐_2014.docx]

各位代表:

现在，我代表承德市人民政府向大会作工作报告，请予审议，并请市政协委员和列席会议的同志提出意见。

一、2013年工作回顾

过去的一年，面对极为复杂严峻的发展形势，我们认真贯彻党的十八大和十八届二中、三中全会精神，全面落实省委八届五次全会和市委十三届四次全会的战略部署，紧紧围绕建设国际旅游城市总目标，按照“发挥优势、高点起步、开放创新、绿色崛起”的总要求，积极应对经济下行和转型升级的双重压力，着力稳增长、调结构、促改革、惠民生、优环境，抢抓机遇，迎难而上，较好地完成了市十三届人大一次会议确定的目标任务。

经济发展稳中向好。全市生产总值达到1272亿元、增长9.3%，增速居全省第2位,一产、三产比重分别提高1.1和0.7个百分点；全部财政收入占GDP的比重达15.1%、比上年提高0.2个百分点，公共财政预算收入102.3亿元、增长23.9%，增速居全省第2位；全社会固定资产投资完成1227亿元、增长20%，增速居全省第4位，一、二、三产投资分别增长31.7%、5.3%、32.7%；万元生产总值能耗下降3.89%，化学需氧量、二氧化硫、氨氮和氮氧化物排放量均完成省下达的目标任务。有5个县财政收入超过10亿元。全市在建亿元以上项目862个，完成投资1070亿元，增长19.5%。双峰寺水库大坝主体工程建设顺利，承德民用机场全面开工，丰宁抽水蓄能电站开工建设，京沈客专具备开工条件。承赤高速竣工通车，中心城区高速环线实现贯通，高速公路通车里程达到552公里。承张高速、张唐铁路加快建设，承平、承锡高速前期工作扎实推进。南郊热电项目获得核准。钒钛基地项目得到国家支持。社会消费品零售总额达到392.6亿元，增长13.5%，成功列入全国集中连片推进农产品流通和农村市场体系建设试点市。外贸出口增长63.5%。

工业转型步伐加快。规模以上工业增加值完成528亿元，增长11.9%，增速居全省第2位。投入各类资金387亿元，实施技改项目495个，新增规上工业企业101家。整合减少矿山企业69家，55钒铝合金产品填补国内空白，实施尾矿资源综合利用项目52个，北汽福田发动机部件铸造中心项目一期竣工投产。列入国家资源型城市可持续发展规划和全国老工业基地调整改造规划。壮大战略新兴产业，风电装机206.7万千瓦，光伏发电发展迅速，承德县中电投一期2万千瓦、平泉县三峡新能源3万千瓦光伏发电项目并网运行。培育认定高新技术和科技型中小企业306家，高新技术产业增加值增长13%。全面开展大气污染防治，实施节能减排项目142个，市区二级以上天数249天，大气质量保持京津冀地区前列，饮用水源地水质达标率保持100%，被全球网民推荐为“最中国生态城市”。争创国家生态文明建设试点示范市取得阶段性成果，被确定为“生态文明示范工程试点”。用地管理不断加强，被评为全国土地“双保工程”先进市。

农业基础得到巩固。粮食产量147万吨，增长9%。蔬菜、食用菌产量分别增长6%和15.6%，新增经济林28.6万亩、中药材8.5万亩，生猪、肉鸡、肉牛、奶牛规模养殖比例分别达到65%、97%、30%和100%。实施千万元以上产业化项目172个，农业产业化经营率达65.4%。杂粮批发市场列入国家规划。实施“五小水利”工程2496处，新增和改善灌溉面积24万亩，治理水土流失面积513平方公里，完成造林绿化82万亩。深入实施新一轮扶贫攻坚规划，投入各类扶贫资金20.4亿元，农村10万贫困人口实现稳定脱贫。

文化旅游深度拓展。开发文化创意、温泉滑雪和康体养生等新兴业态，推出冰上嘉年华、皇家灯会等冬季旅游新产品。高标准、高质量推进山庄外庙文化遗产保护工程，累计开工88项、完工58项。在全省率先创建成为国家级旅游标准化示范城市和国家文化与科技融合示范基地，“热河生物群古生物化石地”保护项目获国家批准。新增鼎盛王朝等省级以上文化产业示范基地5个。成功举办承德国际旅游文化节、中国老年合唱节等节事活动，避暑山庄与俄罗斯圣彼得堡夏宫缔结友好景区。我市参与摄制出品的电视剧《打狗棍》成为宣传承德的一张新名片。接待游客2463万人次，旅游总收入突破200亿元，分别增长22%和26%。

城乡面貌持续改善。编制完成承德市历史文化名城保护等10项重点规划。深入开展中心城区改造整治专项行动，投资8.9亿元综合改善旧小区14.3万平方米，对125个小区、215栋住宅楼公共管网等设施进行专项改造，创建示范路13条，治理街巷81条，拆除违章建筑5.3万平方米，实施10项亮化和22项绿化工程。四道沟至碾子沟、市委党校路竣工通车。太平庄污水处理工程顺利推进，总长11.2公里的9条污水管道全部完工，消除武烈河污水直排口17个，中心城区污水管网实现全覆盖。配建居民小区停车位6000个，新建和改造公厕51个。省级卫生城市通过复审验收。实施县城建设千万元以上项目39个,10个重点镇和150个幸福乡村建设稳步推进，启动了607个村的改造提升工程。平泉通过国家级园林县城验收，新增丰宁、兴隆两个省级园林县城。城镇化率达到43.5%。

改革开放扎实推进。强化综合治税和预算管理，稳步开展“营改增”试点。交通银行落户我市，承德银行进入全国城商行综合竞争力排名前四强，农村信用社改革走在全省前列，成功发行全省首支私募债券和我市首支中小企业集合票据，培育中小企业“新三板”上市融资取得重大进展，与省建行、省交行和东吴证券签署战略合作协议。银行业金融机构存贷款余额分别增长17.8%和14.2%，外埠银行业金融机构贷款余额增长13.7%。组织参加廊坊经贸洽谈会等系列招商活动，成功举办京承合作协调小组第四次会议、北京园区招商合作说明会和承德国际经贸洽谈会。承平高速、津承铁路等18个合作事项分别列入河北省与京津两市合作框架协议，滦平县率先开通直达北京的城际公交。筹建中国节能环保研究院，与北师大、天坛医院等教育医疗机构建立合作关系，微软IT学院进驻承德，对接央企京企137家，合作项目213个，开工建设70个。理顺高新区和3个省级产业示范（聚集）区管理体制，省级以上各类园区达到19家。承德海关办公楼完成主体工程建设。引进市外到位资金470亿元，增长44%。民营经济增加值完成780亿元，增长11.1%。

民生保障更加有力。民生支出占公共财政预算支出的72%，向社会公开承诺的民生事项全部落实。城镇居民人均可支配收入20637元，农民人均纯收入6226元，分别增长10.3%和12.3%。城镇新增就业4.5万人，城镇失业人员再就业3.4万人，农村富余劳动力转移就业4.1万人，城镇登记失业率3.5%，居民消费价格涨幅2.4%。城乡居民社会养老保险参保率达到98.5%，城乡低保补助标准超出全国平均水平。建设保障性住房8760套，竣工1.8万套，改造农村危房2.6万户。27.3万农村人口饮水安全问题得到解决。新建改建标准化菜市场和农贸市场21个、社区便利店和农家店120个，农家店村级覆盖率达到90%，市区平价蔬菜直营店达到30家。优质教育资源覆盖面不断扩大，学前三年毛入园率达到74%，在标准化小学、初中就读的学生分别达到76%和92%，中小学校安工程走在全省前列，普通高考成绩有了新提高。医改各项目标顺利实现，国家基本药物制度全面实施，村卫生室标准化建设实现全覆盖。城镇基本医疗保险参保率和新农合参合率分别达到96.5%和96.2%。人口计生工作继续保持全省先进。“两馆一站”全部免费开放，新增国家级文物保护单位9处，累计达到22处。全民健身运动广泛开展，实施省级以上健身工程55个。平安承德建设深入实施，刑事案件和治安案件发生率明显下降，森林草原防火、安全生产和食品药品安全监管力度加大，人民群众生命财产安全得到有效保障。

政府自身建设不断加强。严格落实中央“八项规定”，清理公务用车和办公用房，“三公经费”支出比上年压减5%以上。深化行政审批制度改革，取消和清理审批事项105项。市政务服务中心投入使用，市县区公共资源交易中心全部建成。强化审计监督和行政监察，加强工程建设、土地和矿权出让等重点领域监管。自觉接受人大的法律监督、工作监督和政协民主监督，办理市级以上人大代表建议83件、政协委员提案221件，办结率均达到100%。

国防动员、双拥共建深入开展，军政军民团结局面进一步巩固，对口援疆、民族宗教、广播电视、外事侨务、防震减灾、邮政通信、妇女儿童、人防、气象、统计、档案、老龄、残疾人等事业取得新成绩。

各位代表，过去的一年，面对各种困难挑战，我们牢牢把握发展第一要务，咬定青山不放松，全市上下形成了跨越赶超的强烈共识；我们主动适应新形势，发挥优势，抢抓机遇，全力推动生态文明与经济社会协调发展，使区域战略层级明显提升；我们坚持高点起步，合力攻坚，着力推进基础设施建设、产业结构调整，一批打基础、利长远的重大项目取得历史性突破；我们掷地有声抓落实，像锤子钉钉子一样解决问题，汇聚了谋事干事、善作善成的正能量；我们坚持底线思维，创新社会管理，化解各种矛盾，巩固发展了社会安全稳定的大好局面。

各位代表，过去一年的成绩来之不易，这是党中央、国务院和省委、省政府亲切关怀、大力支持的结果，是中共承德市委科学决策、正确领导的结果，是全市人民同心同德、拼搏进取的结果。在此，我代表市人民政府，向全市人民，向人大代表、政协委员，向各民主党派、工商联、无党派人士和人民团体，向驻承人民解放军、武警官兵和政法干警，向一直以来关心和支持承德发展的各界朋友，致以崇高的敬意和衷心的感谢！二、2014年工作要求和目标任务

今年是贯彻落实党的十八届三中全会精神、全面深化改革的开局之年，是实施“十二五”规划、全面建成小康社会决定性阶段的重要一年。新的一年，国家和省将陆续出台一系列改革措施，必将极大地激发我市经济社会发展的内生动力；京津冀协同发展和首都经济圈规划上升为国家战略，北京启动实施功能疏解，必将为我们加快发展带来前所未有的机遇；省委、省政府全力实施“四大攻坚战”，重点支持我市基础设施和生态建设，尤其是新一轮扶贫开发规划的深入实施，必将为我们加快发展赢得更多支持；承德众多市场主体、各界人士涌动着创业激情，外界有识之士投资承德的愿望强烈，全市各级党政组织和领导干部更有带领人民群众干事创业的担当，必将为我们加快发展增添新的活力、动力和竞争力。

同时也要看到，尽管去年大多数目标任务完成或超额完成，但仍有生产总值、固定资产投资、全部财政收入等几项指标低于年初预期。今年的发展，还面临着诸多困难和挑战。一是转型升级任务重。单纯以矿山采选和黑色冶金压延业支撑发展的局面还没有根本改变，抵御市场风险的能力较弱。第三产业比重低、发展慢，与国际旅游城市的定位还不相称。二是节能减排压力大。我市虽然生态环境较好，但经济总量小，产业基础薄弱，随着工业技改和节能环保工作力度的不断加大，与其他市同比例降低能耗和减少污染排放的空间较小。三是农民持续增收难。新型农业经营体系还不健全，农业生产经营规模化、集约化程度不高，农民财产性收入低，贫困人口多，扶贫攻坚任务艰巨。四是城镇带动能力弱。产城融合度低，历史欠账较多，城乡基础设施滞后，城市辐射承载能力不强。五是开放面临新挑战。当前环首都各市都在竞相抢抓北京功能疏解和产业转移的机遇，而且国家将出台统一规范的招商引资政策法规，这对于发展基础薄弱、开放度本来就低的承德来说，将面临着既想引进大项目、好项目，又付不起巨额引资成本的两难选择。对此，我们既要保持清醒头脑，做到未雨绸缪，也要树立信心勇气，牢牢把握工作主动权。

今年政府工作的总体要求是：认真贯彻党的十八届三中全会精神和省委八届六次全会、市委十三届五次全会部署，把握环渤海加速发展和京津冀协同发展的重大机遇，按照“发挥优势、高点起步、开放创新、绿色崛起”的总要求，坚持稳中求进、进中求好的主基调，以建设国际旅游城市为总目标，以创建国家级生态文明与经济社会协调发展试验区为总抓手，着力推进改革创新，着力推进转方式调结构，着力推进城乡一体化发展，着力保障和改善民生，坚定不移走绿色循环低碳节能环保发展之路，努力建设美丽幸福承德。

今年经济社会发展的主要预期目标是：全市生产总值增长9%左右；公共财政预算收入增长10%；全社会固定资产投资增长19%；规模以上工业增加值增长11.5%；社会消费品零售总额增长13%；单位GDP能耗下降3.89%，化学需氧量、二氧化硫、氨氮和氮氧化物排放量分别削减2.2%、2%、2.5%和1.8%；城镇居民人均可支配收入和农民人均纯收入分别增长10%；城镇登记失业率控制在4.5%以内；居民消费价格涨幅4%左右；人口自然增长率控制在7.6％。以内。

实现今年经济社会发展目标，必须全力以赴抓好“改革创新、生态优先、转型升级、开放发展”四大任务，以重点突破活跃带动全局。改革创新，就是牢牢把握全面深化改革这一根本动力，把改革创新贯穿于经济社会发展的各领域各环节，以改革创新促发展、促转方式调结构、促民生改善。生态优先，就是牢牢把握生态这个可持续发展的第一资源，探索建立生态优势转化为发展优势的有效途径，走出一条“产业生态化、生态产业化”的绿色崛起之路。转型升级，就是牢牢把握产业转型升级这一主攻方向，改造提升传统产业，大力培育新兴产业，加快构建现代产业体系，推动经济提质增效。开放发展，就是牢牢把握扩大开放这一基本途径，顺应区域经济一体化发展大势，紧盯首都北京，大力引进优质资源和高端要素，抢占发展先机，打造新的竞争优势。

三、坚定不移全面深化改革

改革是经济发展和社会进步的永恒动力。我们要坚持用创新的思维、改革的办法，充分发挥市场在资源配置中的决定性作用和更好发挥政府作用，从制约经济社会发展的突出问题和群众最期盼的领域入手，正确、准确、有序、协调推进改革。

（一）深化行政管理体制改革。进一步简政放权，承接落实好国家和省下放取消的行政审批事项，全面清理市本级行政审批事项，把该放的放下去、放到位，把该管的管得住、管得好。启动市县政府机构改革，推进政府机关标准化建设，推行政府向社会购买服务。加快构建更大范围的网上审批和监管平台，最大限度地减少和规范自由裁量权。开展县向乡镇直接放权试点，增加基层干事的权力。稳步推进事业单位分类改革。加快政府与社会组织分离脱钩，促进行业协会、商会等中介组织健康发展。

（二）着力清除各种市场壁垒。创新国有资产管理体制，探索组建不同类型的国有资本运营管理平台。按照非禁即允、非限即许的原则，放开民间投资领域，逐步实行负面清单管理，鼓励民营经济参与国企改革、进入特许经营领域、控股混合所有制企业。推进商事登记改革，推行投资主体“零限制”、注册登记“零收费”，实行先照后证登记制、注册资本认缴制、年度报告公示制，加快形成“宽准入、优服务、无障碍”的市场主体准入机制。鼓励支持民营经济发展，落实政策措施，完善中小企业社会化服务体系，推动全民创业和民企二次创业，民营经济增加值增长12%以上。

（三）加快推进农村综合改革。围绕农业要强、农村要美、农民要富的目标，认真贯彻落实中央和省深化农村综合改革的一系列政策措施。推进农村土地制度改革，落实集体所有权、稳定农户承包权、放活土地经营权，完成农村土地确权登记，探索建立综合性农村产权交易机构，促进农村产权公开、公正、规范交易流转，新增土地流转面积10万亩以上，累计达到80万亩。探索建立城乡统一的建设用地市场，推动集体经营性建设用地和国有建设用地同市同权同价。创新农业生产经营方式，培育新型经营主体，提高规模化经营、集约化发展、社会化服务水平，推广双滦区肖店村经验，抓好集体经济股份合作制试点。进一步强化乡镇村级管理和服务功能，鼓励在外的退休干部、企业家和务工人才回村创业。

（四）积极推进财税金融改革。完善政府预算制度，早编细编预算，探索城建复式预算管理办法。强化依法治税，坚决制止越权或变相减免税收行为。建立政府性债务偿还联席会议制度，扩大偿债准备金来源和规模，积极稳妥做好政府性债务管控和化解工作。深入研究“五大集团”规范有效运营。积极探索和鼓励引导民间资本依法设立中小型银行等金融机构。推进企业在多层次资本市场上市融资，确保天宝矿业在香港上市，力促兴安民爆在境外上市，争取10家以上中小企业集中在“新三板”挂牌，每个县确保有1个上市公司。深入实施“引银入承”工程，引进北京、成都等农商行来我市领办村镇银行，力争四大国有银行县域机构全覆盖，推动承德农商行组建，支持承德银行加快发展。全市新增贷款140亿元以上。

（五）扎实推进社会事业改革。深化教育领域综合改革，统筹城乡义务教育资源均衡配置，保障进城务工人员随迁子女平等接受义务教育，鼓励社会力量兴办教育。建立城乡统一的教育编制动态调整和管理机制，推动学校“去行政化”。研究组建文化旅游资产专门管理机构，统筹管理、综合开发资源，实现资源资产的保值增值。深化医药卫生体制改革，扩大居民大病保险试点，全面推开县级公立医院改革，探索乡镇卫生院县乡一体化管理，鼓励社会资本办医，有序扩大基本药物制度实施范围。研究制定一方是独生子女的夫妇可生育两个孩子的具体实施办法。

四、生态优先增创竞争优势

生态是承德可持续发展的第一资源。作为京津的生态屏障和重要水源地，保护生态环境既是承德的“命门之穴”，更是培育发展优势的内在要求。我们要以争创国家生态文明与经济社会协调发展试验区为抓手，充分发挥生态第一竞争力的作用。

（一）全力开展大气污染防治。把保持环京津地区大气质量最优作为底线，集中开展大气污染防治攻坚行动，压减炼铁产能30万吨，提前一年完成“十二五”淘汰落后产能任务。加快淘汰城区燃煤锅炉，削减煤炭用量87万吨。积极推进绿色施工，严格限制露天开采，铁路公路两侧、旅游景区景点可视范围内的矿山逐步有序退出。推行道路机械化清扫等低尘作业，市区和县城机械化清扫率分别达到50%和40%。加快淘汰“黄标车”。开展化工、制药等行业挥发性有机物治理，完成油气回收治理工程，严控油气烟气排放，禁止露天餐饮和焚烧秸秆。抓好循环经济“双五十百千”工程和污染减排工程。推进集中供热覆盖、天然气替代以及其他清洁能源应用。

（二）强力推进水环境治理。全面实行“河长制”，严格落实水污染排放标准。以武烈河为重点，对滦河、潮河等水系进行综合治理，全力抓好河道垃圾清理、违法排污口取缔、污水管网改造、矿山废弃水治理。市污水处理厂一期改造、二期扩能和上板城白河南污水处理厂建成使用。加强饮用水源保护区环境管理，逐步建立地下水质量监测网络，提高环境准入标准，预防地下水污染，城市集中式饮用水水源地水质达标率保持100%。加快农村饮水工程建设，解决25万农村人口饮水安全问题。

（三）大力实施重点生态工程。把涵养和修复生态作为首要任务，重点实施京津风沙源治理二期、21世纪首水、再造三个塞罕坝、三北防护林、京冀水源林等生态工程，启动新一轮退耕还林工程，提高国土绿化水平。深入推进百矿披绿、土地复垦、花园式选厂等工程，严格限制“开山”搞建设。新增造林绿化70万亩，治理水土流失面积500平方公里。积极推进“千湖工程”，加快在建水利项目建设，新建一批“五小水利”工程，提高水资源保障和控制能力，保持生态环境质量华北领先。

（四）着力推动生态文明制度建设。从具体制度设计入手，探索利用市场机制推动资源转化变现的有效途径，强化对绿色发展的刚性约束。按照主体功能区划要求，合理确定产业发展布局、结构和规模，实施更加严格的环境准入标准。加大环境执法力度，坚决依法打击各类环境违法行为。健全排污权交易机制，完善自然资源资产产权制度，推进生态产品总值评估工作，探索建立水资源和碳汇、节能量交易所等市场平台。以中国节能环保研究院为依托，推行生态环保管理“第三方治理”模式。加快形成生态补偿的市场机制，构建与京津生态建设保护的互动机制。五、调整结构加快产业转型升级

坚持立足优势，瞄准高端，市场引领，创新驱动，绿色低碳，以打造千亿元产业为抓手，做好传统产业“有中生新”和新兴产业“无中生有”文章，加快构建现代产业体系，推动经济提质增效升级。

（一）优先发展高端旅游服务业。实施“七大工程”行动计划，加快滦平金山岭长城、隆化七家——茅荆坝温泉、围场御道口至尊、兴隆燕山国际、承德县唐家湾等重点旅游休闲度假区建设。金山岭长城景区争取通过5A验收，七家行宫一期、王府世家滑雪场建成投用，凤凰谷生态园区和御道口滑雪场开工建设，各县区建成一个重点旅游项目、开工一个冬季旅游项目。建设精品旅游环线、精品旅游景区、乡村旅游度假带，完善旅游景区标牌、道路、公厕、停车场等基础设施，增强吃住行游购娱要素保障能力。抓好“智慧旅游”建设和市场秩序整顿，加大旅游宣传推介力度，组织举办第14届中国承德旅游文化节、冰雪欢乐汇和“两岸观光博览会”等重大活动。启动创建国家级旅游综合改革试点，建立旅游营销联盟，拓展境外客源市场。接待游客和旅游总收入分别增长19%和26%。

（二）加快壮大先进装备制造业。以发展节能环保、智能化仪器仪表、汽车零部件、自动输送机仓储、矿山采掘、石油机械等装备制造业为重点，向精品化、系统化、高端化、集团化方向发展，实现主营业务收入170亿元，利税20亿元。北汽福田精密铸造产能达到10万吨，力争新能源汽车、精加工、特种车制造项目落户我市。平泉航空科技产业园争取两家以上新企业和通用航空维修保养、培训等项目入驻。兴隆钢研产业园完成起步区七通一平。苏垦银河连杆新增190万件生产能力。

（三）积极培育高新技术产业。加强与北京科研院所、高等院校的科技合作，建设环首都科技成果孵化园区，争创省级重大创新基地。加快推进中国节能环保、中国发动机工艺、中国遥感技术、中国特种车辆、中国钒钛等5家研究院建设，推动建设承德国家级大型数据中心。重点实施燃烧合成氮化钒铁、航天级钒铝合金、钒电池试验示范、锂离子电池研发等一批科技支撑项目，培育科技型亿元以上小巨人企业5家、中小企业50家，力争更多企业入选省千家科技型中小企业，规模以上高新技术产业增加值增长13%以上。

（四）做大做强钒钛新材料产业。拉长产业链条，整合矿产资源，引进战略投资者，力促钒钛基地新厂开工建设，支持金科科技氮化钒铁等高端产品扩大规模，钒钛新材料产业销售收入突破1000亿元。充分利用尾矿资源，抓好微晶板材试验、彩色玻璃装饰板研发等“十大科研课题”攻坚，制定铁尾矿混凝土应用技术标准，加快筹建省级尾矿工程技术研究院，建设2个综合示范区、3个尾矿新型建材园区，尾矿开发利用企业达到80家以上，产值突破100亿元。

（五）大力发展清洁能源产业。抓住省支持发展规模化光伏电站的机遇，全力打造千万千瓦级风电、千万千瓦级水电调峰电源、千万千瓦级太阳能发电三大清洁能源基地。加快丰宁抽水蓄能电站一期工程建设，争取华御围场风光一体化、中电投承德县光伏二期8万千瓦等一批重点项目建成投运。光伏发电装机达到25万千瓦，风电装机260万千瓦。争列国家生物质能（木煤）供热示范区，木煤生产能力65万吨。天然气用户达到1万户以上。清洁能源实现产值65亿元。

（六）提升发展食品药品加工产业。大力发展现代农业，建园区、扩基地、育龙头，打造“绿色、有机、安全”品牌，建设首都绿色有机农产品生产加工基地。每个县建设现代农业园区、家庭农场、股份制合作公司分别达到3个以上。新建和改造千亩以上现代蔬菜产业园区21个，争列10万亩以上省级蔬菜大县5个，新栽和改造经济林100万亩，新增中药材5万亩、食用菌2.3亿盘，支持丰宁缘天然奶业做大做强。实施千万元以上农业产业化项目150个，产业化经营率达到66%以上，新增河北省名牌产品5个。鼓励中药产品研发生产，力争规模以上企业达到10家，实现销售收入25亿元。

（七）支持发展现代商贸物流产业。借助综合交通体系和产业聚集区，大力引进知名物流企业，培育一批以运输、仓储、装卸、加工、整理、配送、信息等功能为一体的现代物流配送中心，主营业务收入超5亿元的大型物流企业达到3家。鼓励规模特色产业、大宗商品交易到园区开办专业市场，建设交易规模超5亿元的大型流通市场4个。双滦国际商贸物流园区销售收入达到50亿元，平泉华北物流园建成区面积达到2.3平方公里，丰宁现代物流园区完成投资5亿元以上，承德空港物流园完成园区规划。

（八）积极开拓文化创意产业。紧紧抓住建设国家文化与科技融合示范基地的机遇，推进文化创意与旅游的深度融合，重点培育皇家、佛教、草原及民族民俗等文化产业园区，鼓励支持腾氏布糊画、满族剪纸、活性炭等特色文化艺术品的研发生产。大力发展网络动漫、数字娱乐、设计服务等新兴文化产业，推动星月5D科技文化产业基地项目签约落地，培植文化创新企业30家。每个县区培育1个特色优势文化产业，形成1个知名文化品牌。

六、面向京津扩大开放合作

把握京津冀协同发展的大势，全方位、深层次、多领域扩大开放合作，努力提高对内对外开放水平。

（一）主动对接融入京津。把京津尤其是北京作为对外开放的主攻方向，落实京承合作协调小组会议确定的重大事项，跑部进京、深度对接，力争更多专项列入《首都经济圈发展规划》。加强与中国建材、大唐国际、航天科技、保利集团、首农集团等央企京企的合作，对接央企京企50家以上。积极探索医疗、教育、就业、人口等方面跨市顺畅衔接的可行机制，力争让更多优质资源和高端要素为我所用，北京师范大学承德附属学校实现暑期招生。依托生态、温泉等旅游资源，建设特色养生小镇、温泉水城、山庄人家，建成一批示范性度假村和养老基地，打造首都经济圈高端人群疗养度假的休闲区。

（二）拓宽开放合作视野。瞄准市场需求和产业发展方向，有选择地引进项目和企业，实现错位发展、优势互补。抓住南资北移、台资北移、外资北移的机遇，引进长三角、珠三角地区的人才、资金和项目，鼓励建设深圳产业园、江浙产业园、台商产业园，实现以企引企、以商招商。积极发展对外贸易，瞄准世界500强企业和国际先进技术，扩大对外经济合作和利用外资规模，引进市外到位资金550亿元,实际利用外资1.45亿美元。

（三）建好项目“落地”平台。把园区作为承接产业转移的重要载体和支撑，落实既有政策措施，创新管理体制机制，提升配套功能，统筹推进以“1+3”产业发展平台为引领的园区建设，实施高新区产业倍增三年推进计划，3个省级聚集（示范）区的起步区加快完成“七通一平”，中关村承德创新创业大厦、张百湾、六沟聚集区综合服务中心开工建设，引进亿元以上项目87个。力争建成1个中外合作开发园区。扎实做好县区经济开发区的整合工作，每个县至少有1个省级经济技术开发区。

（四）增强招商引资实效。建立和完善与北京全方位对接沟通机制，做好点对点、小组团招商，努力提高履约率。设立不少于3000万元的招商奖励基金，按投资标准给予项目引荐人和项目小组奖励，调动全民招商的积极性。组织参加香港投洽会、厦门投洽会等活动，办好承德国际经济贸易洽谈会，建设承德招商、欧洲招商展示中心和义乌承德特色商品展厅，打造品牌、扩大影响、增强实效。

七、项目牵引推动经济持续发展

发展是解决所有问题的关键。我们要始终坚持发展这个第一要务，把握关键，强化支撑，挖掘潜力，保持经济持续稳定增长的良好态势。

（一）充分发挥项目投资的关键作用。牢固树立抓项目就是抓发展的理念，继续实施项目包保责任制，建立评估论证、资源配置、前期经费、强化奖惩等工作机制，全力以赴跑前期、保落地、抓储备，确保实现“十百千万”目标。加快承津、承秦铁路和围场通用机场等重大项目的前期工作，争列省重点50个；推进承德民用机场、双峰寺水库、南郊热电等项目建设，力促京沈客专承德段、承平、承锡高速公路开工，虎蓝铁路、张唐铁路具备通车条件，市级重点项目达到100个、超亿元项目1000个；紧跟国家投资方向和重点，在节能环保、生态建设、改善民生等领域谋划一批大项目好项目，储备入库项目达到1万个以上。

（二）充分发挥扩大消费的基础作用。培育壮大商贸流通行业市场主体，抓好10大商业综合体、6条特色街区、4家星级酒店的建设和招商。推进“万村千乡”市场工程和农超对接，改造提升农村集贸市场60个。建设名优农副产品展示展销中心，筹建电子商务园区，新建淘宝网“承德购”等电子商务平台5个以上。深入开展住宿餐饮业提升行动，办好美食文化节。规范房地产市场健康发展，鼓励本地房地产企业拓展以北京为重点的外埠市场。实施城乡居民收入倍增计划，提升居民即期消费能力。新增出口备案企业10家，外贸出口达到2.4亿美元。

（三）充分发挥骨干企业的带动作用。对入选省“三个一百”企业进行重点扶持，推动工业“50强”扩规提质，力争销售收入10亿元企业达到25家以上，着力培育10个百亿元企业集团。实施天大钒业、华远自动化、金建检测仪器等投资3000万元以上技改扩能项目250个，其中投资亿元以上的100个，力争露露扩能竣工投产。对成长性好的企业进行重点帮扶，支持小巨人企业向大企业迈进，扶持小微企业提档升级，新增规上工业企业100家以上。完善企业用电、用气应急预案，在融资、用地、生产资料等方面给予支持。组织开展好第三次经济普查。

（四）充分发挥县域经济的支撑作用。依据省市重大战略，分类指导各县区加快发展。支持滦平县以张百湾新兴产业示范区为依托，积极承接北京产业转移；支持丰宁县扩大与金融企业的合作，加快发展奶业和木煤等绿色循环产业；支持兴隆县加快京沈客专、承平高速等重大基础设施建设，提高保障发展的能力；支持承德县与市区基础设施对接，加快同城化步伐；支持隆化县重点园区建设，发展装备制造、休闲度假等特色产业；支持围场县发展生态旅游、大数据产业和特色农业，开展生态文明与经济社会协调发展试点；支持平泉县加快统筹城乡和农村综合改革，推进撤县建市；支持宽城县整合提升冶金矿山企业，促进矿业经济转型。支持高新区深化改革，创新管理，打造城市产业发展高地；支持双桥区抓好山庄外庙周边综合开发和北部新城建设，提升服务业水平和承载能力；支持双滦区依托文化产业和物流园区建设，重点发展现代服务业；支持营子区依托建龙特钢发展钒钛产业，推动资源枯竭型城市转型发展。

（五）充分发挥扶贫攻坚的助推作用。抓住国家和省对革命老区、民族地区、边疆地区、贫困地区增加转移支付的机遇，深入实施燕山——太行山片区和环首扶贫攻坚示范区规划，按照贫困村3年部分脱贫、5年基本脱贫的要求，创新机制，增加投入，在精准扶贫、产业扶贫、金融扶贫、社会扶贫、行业扶贫上下功夫。抓好“大园区、小业主”试点，推行“五位一体”扶贫模式，加大新型农民培训力度，大力发展股份合作制经济、新型家庭手工业和现代农业园区。采取产业扶贫和移民搬迁相结合，做大做优特色产业，力促贫困村向新农村转化。各类扶贫投入达到25亿元以上，确保10万贫困人口稳定脱贫。八、积极稳妥推进新型城镇化

城镇化的核心是人的城镇化。加快城乡一体化、公共服务均等化，推动中心城市、县城和小城镇协调发展，努力走出一条以人为本、优化布局、生态文明、传承文化的新型城镇化道路。

（一）提升中心城市承载带动能力。围绕建设“服务首都特色功能城市”，进一步明确城市功能定位，坚持把城市组团作为城市发展的基本形态，优化发展布局，推进城市总体规划修编，整合公共服务设施规划，完成城市门户和重要节点的城市设计。继续推进老城区中疏，限制高度、疏减密度、控制强度，杜绝大拆大建，继续实施中心城区综合改造整治工程，推进工业和商业批发企业外迁，加快回迁房建设，抓好17项绿化工程和一批重点亮化项目，完善旅游标准化导引系统建设，创建丽正门大街、武烈路精品示范路。做强南部新城，打造冯营子高铁商圈和闫营子、大石庙商务中心，完成滨河新城中小学、三甲医院等配套设施建设。做优北部新城，引进知名企业，谋划实施一批文博会展、体育休闲、影视基地等高端服务项目。大力发展绿色节能建筑，开工建设“被动式—低能耗”住宅小区项目。完善城市网格化管理机制，提高综合管理水平。

（二）加快发展特色县城和小城镇。坚持科学规划，体现尊重自然，突出地域特色，延续历史文脉，让居民望得见山、看得见水、记得住乡愁。抓好县城建设，实施一批公共服务、园林绿化、景观风貌等方面的重点工程，抓好平泉、兴隆两个省级县城试点县建设，创建滦平、宽城两个国家园林县城和营子、隆化两个省级园林县城。突出乡镇特色，打造一批文化古镇、商贸新镇、经济强镇和旅游名镇，抓好29个重点乡镇建设。按照增加现代设施、保持田园风光、传承优秀文化、绿化村庄庭院的要求，抓好325个村的农村面貌改造提升，开展秸秆资源化利用，推进农村太阳能分布式发电，彻底消灭“连茅圈”，促进农村人居环境明显改善。坚持产城融合，走就地城镇化、农民变市民的路子，全面放开县城和建制镇的落户限制，有序推进农业转移人口市民化。城镇化率提高1.5个百分点。

（三）统筹城乡基础设施建设。围绕加密路网、打造水网、建强电网、调整热网，加快推进城乡基础设施一体化。投资33亿元续建新建干线公路620公里，完成314个村主街道路硬化任务，全面推进农村公路“田路分家”。投资40亿元，完善高速公路连接线，全面开工建设单塔子至大栅子一级公路，开辟市区第二条迎宾大道，其中牛圈子沟至秫秸沟隧道建成通车，单塔子综合客运枢纽建成投入使用。积极推动北京与兴隆、丰宁间开通城际公交，增开市区至下板城和营子的高速公交、至韩麻营和张百湾的快速公交，开通狮子园路、丹霞路等环城公交，加快城市公交向重点乡镇延伸。实施市区“六条旱河”水环境综合整治工程，东区、南区两座水厂具备供水条件，城市供水普及率达到99.5%。承德东500千伏变电站开工建设。实施中心城区供热保障工程，双桥区新增供热面积60万平方米，双滦区启动40公里管网铺设和供热计量改造工程，五道沟、草庐沟天然气供热项目投入使用。

九、更加注重保障和改善民生

把民之所望作为施政方向，突出重点、完善制度、引导舆论，优先保障民生投入，优先安排民生项目，优先解决民生问题，使发展的成果更多更公平地惠及全市人民。

（一）大力发展社会事业。优先发展教育事业。启动实施第二期学前教育三年行动计划，省市级幼儿示范园达到100所，在标准化学校就读的小学、初中学生分别达到78%和96%，6个县区实现义务教育基本均衡，11个县区全部通过普高验收，国家级示范性中职学校达到3所。改造加固学校443所，购置设备1.27万台（套）。继续实施农村义务教育阶段学生营养改善计划。加快发展卫生事业。优化市区医疗资源配置，市中医院竣工投入使用，市儿童医院开工建设。完成二级综合医院等级评审，启动二级专科医院评审工作。基本药物网采率保持100%、配送率达到90%以上。做好人口计生工作，稳定低生育水平。加强文化事业建设。山庄外庙文化遗产保护主要工程全部完工。市县数字图书馆建成并投入使用，市县图书馆、乡镇综合文化站、农家书屋免费开放。建设滦平县全国普通话体验区。

大力发展体育事业。建设奥体中心综合性全民健身广场，打造50万平方米“两河四带”体育休闲长廊。

（二）办好惠民利民实事。统筹城乡平等就业，城镇新增就业4万人，城镇登记失业率控制在4.5%以内，农村富余劳动力转移就业4万人。提高城乡居民收入，企业退休人员基本养老金提高10%，落实机关事业单位津贴补贴制度改革政策。大力发展非农产业，努力增加农民收入。推进社会保险扩面，新增城镇各类保险参保6万人，城乡居民养老保险参保率稳定在90%以上。城镇基本医疗保险参保率稳定在96%以上，新农合参合率稳定在95%以上、筹资标准提高到390元。城乡居民医疗补助标准提高到320元。保障困难群体基本生活，五保集中供养标准由每年2650元提高到4250元，分散供养标准由每年1980元提高到3800元；建立贫困重度残疾人生活补贴和贫困残疾儿童挽救性康复补贴制度，对一、二级重度残疾人每月补贴50元，开工建设民政事业服务中心、市残疾人康复和托养服务中心。提高养老服务水平，鼓励社会资本兴办养老育幼、托养及家政护理机构，扩大城市居家养老服务中心和农村互助幸福院覆盖范围，力争每千名老人养老床位数达到27张。加大住房保障力度，新建保障性安居住房1.45万套,竣工入住9100套，改造农村危房1.2万户。改善城区居民生活条件，完成75万平方米建筑节能改造，对中心城区大老虎沟15.6万平方米区域小联片实施集中供热与建筑节能改造，拆除该区域内10座燃煤锅炉，彻底解决1500户居民集中供热问题。建设便民服务设施，打造一刻钟便民生活圈，建成市区家政服务网络中心。新建社区便利店50个、小区便民市场3处，完成桥东市场改造，南区农产品批发市场建成使用，改造农村集贸市场60个。配建小区停车位4000个以上。

（三）维护社会和谐稳定。严格落实安全生产责任制，加强矿山、尾矿库等重点行业、重点领域监管，有效遏制各类安全事故的发生。做好森林草原防火和防汛抗旱等工作，提高技防监测水平和防灾减灾能力。理顺食品药品监管体制，深入开展食品药品隐患排查和专项整治，以“四个最严”为标准，确保人民群众“舌尖上的安全”。深化以“四有一创”为主要内容的社区建设，实现社区网格化管理服务全覆盖。创新社会治安综合治理模式，健全“三位一体”综合调解机制，努力化解社会矛盾，保持社会大局和谐稳定。

十、转变职能加强政府自身建设

围绕建设法治和服务型政府，推进政府治理体系和治理能力建设，不断提高政府的公信力和执行力，为转型升级、绿色崛起提供坚强保障。

（一）坚持为民执政。深入开展党的群众路线教育实践活动，从群众最关心的事情干起、从群众反映最强烈的问题改起、从群众意见最集中的地方抓起，切实解决热点难点问题，让群众看到实实在在变化、得到更多实惠。深入开展调查研究，精简会议文件，坚决整治不作为、慢作为、乱作为。加强政务服务中心建设，落实首问负责制、限时办结制和领办代办制，实行“零障碍”全程协办服务，做到“一次告知、两次办结”，真正办成“市民服务中心”。开通运行961890服务热线，24小时受理群众诉求，不断提高政务服务水平。

（二）坚持依法行政。把遵守宪法和法律作为施政的根本准则，严格按照法定权限和程序行使权力、履行职责。推进行政权力公开透明运行，切实做到有法必依、执法必严、违法必究，努力在制度上为市场主体和人民群众营造公平竞争、公平发展的机会，促进社会公平正义。认真接受人大的法律监督和工作监督，自觉接受政协民主监督，重视发挥新闻舆论和社会公众的监督作用，保障人民群众的知情权、参与权、表达权、监督权。

（三）坚持科学理政。注重系统谋划，强化顶层设计，用新举措破解难题，以新思路引领发展。健全科学民主决策机制，完善重大行政决策程序，加强行政决策合法性审查和风险评估。坚持集体决策和个人分工负责相结合，做到充分授权，权责匹配，对重大发展战略、重大项目安排、重大工作部署进行充分论证，广泛听取意见，对决策过程、决策实施实行全方位监督，最大限度避免风险和失误。坚持问政于民、问需于民、问计于民，积极采取听证会、网上征询等方式，推动公众参与决策，保障决策更加科学、切合实际。

（四）坚持廉洁从政。认真落实中央“八项规定”，坚决抵制和克服“四风”，牢固树立过紧日子思想，厉行勤俭节约，反对铺张浪费，严格办公用房、用车、会议、接待、出差等管理，严控“三公经费”支出，大幅降低行政成本。加强行政监察和审计监督，强化对财政资金使用、重大项目投资、工程招标、政府采购、国有资产资源经营管理等重点领域监管，最大限度发挥国有资金、资产、资源效益。严格落实党风廉政建设责任制，完善惩治和预防腐败体系，加强对权力运行的制约和监督，做到干部清正、政府清廉、政治清明。

各位代表，改革开启新征程，时代赋予新使命。让我们更加紧密地团结在以习近平同志为总书记的党中央周围，在省委、省政府和中共承德市委的正确领导下，紧紧依靠全市人民，坚定信心，真抓实干，锐意进取，为全面建成小康社会、建设美丽幸福承德而努力奋斗！
